# The Retrograde Frequency Response of Passive Dendritic Trees Constrains the Nonlinear Firing Behaviour of a Reduced Neuron Model

**DOI:** 10.1371/journal.pone.0043654

**Published:** 2012-08-20

**Authors:** Hojeong Kim, Kelvin E. Jones

**Affiliations:** 1 Department of Biomedical Engineering, University of Alberta, Edmonton, Alberta, Canada; 2 Centre for Neuroscience, University of Alberta, Edmonton, Alberta, Canada; 3 Faculty of Physical Education and Recreation, University of Alberta, Edmonton, Alberta, Canada; Neuroscience Campus Amsterdam, VU University, The Netherlands

## Abstract

Our goal was to investigate how the propagation of alternating signals (i.e. AC), like action potentials, into the dendrites influenced nonlinear firing behaviour of motor neurons using a systematically reduced neuron model. A recently developed reduced modeling approach using only steady-current (i.e. DC) signaling was analytically expanded to retain features of the frequency-response analysis carried out in multicompartment anatomically reconstructed models. Bifurcation analysis of the extended model showed that the typically overlooked parameter of AC amplitude attenuation was positively correlated with the current threshold for the activation of a plateau potential in the dendrite. Within the multiparameter space map of the reduced model the region demonstrating “fully-bistable” firing was bounded by directional DC attenuation values that were negatively correlated to AC attenuation. Based on these results we conclude that analytically derived reduced models of dendritic trees should be fit on DC and AC signaling, as both are important biophysical parameters governing the nonlinear firing behaviour of motor neurons.

## Introduction

Action potentials generated near the cell body propagate to the axonal terminals and also retrogradely into the dendritic trees to interact with a number of voltage-gated ion channels (VGICs). In spinal motor neurons from various species (cat [1], [2], [3], turtle [4], [5], rat [6], [7] and mouse [8], [9]), L-type Ca^2+^ channels activated at low voltage and generating persistent inward currents (PICs) have been shown to be the mechanism generating plateau potentials that give rise to nonlinear firing behaviour (e.g. bistable firing) [7], [10], [11], [12], [13], [14]. There is consensus in the community of motor neuron researchers that the calcium mediated PIC channels are located on dendrites about 300–500 µm away from the soma [9], [15], [16], [17], [18]. To date there has been little research on the interaction between the biophysical parameters that govern retrograde propagation of AC signals, like action potentials, and activation of calcium mediated PICs and the resulting plateau potentials. It is important to establish the nature of this interaction, if any, since the plateau potentials give rise to nonlinear firing behaviour in motor neurons, and this in turn has important implications for movement [19].

The dynamics underlying the nonlinear firing of motor neurons is governed by the spatio-temporal interaction between VGICs responsible for plateau potentials in the dendrites, and VGICs responsible for spiking in the soma. This interaction is influenced not only by active properties of the VGICs such as maximum conductance and gating kinetics, but also by signal propagation in the complex dendritic system [20]. In the present study, we focused on how the dendritic signal propagation influenced the nonlinear firing of motor neurons. We previously characterized dendritic signaling properties by measuring voltage attenuation between the soma and all points of anatomically reconstructed dendrites [21]. Our previous work used direct-current (DC) inputs as an analogy for the experimental condition of current step stimulation at the soma and currents generating plateau potentials in the dendrites (i.e. both DC signals). We developed a systematic reduced modeling framework that analytically solved for all passive membrane parameters using empirical measurements of anatomy, input resistance and time constant from mammalian motor neurons [21]. The main result from that model was the finding that direction-dependent voltage attenuation (DDVA) deterministically changed the input resistance in the dendrites altering the firing behaviour of the model to generate all experimentally observed firing patterns, which have been classified into four types (i.e. Type I to IV) based on firing rate (F)-current intensity (I) relationship [2], [6]. Briefly, Type I for linearly overlapped F-I curve without sustained firing, Type II for clockwise F-I curve without sustained firing, Type III for linearly overlapped F-I curve with sustained firing, and Type IV for counter-clockwise F-I curve with sustained firing where Type III and IV (or fully-bistable) firing have been reported to be mediated by PICs generating plateau potentials. In addition to DC signals, alternating-current (AC) signals, like action potentials, may also be involved in nonlinear firing of motor neurons. Action potentials propagate back into the dendrites and may play an important role in the activation of dendritic VGICs. The contribution of dendritic AC signal propagation to nonlinear dynamics of motor neurons, has not been thoroughly investigated, probably due to the lack of suitable theoretical framework to deal with the complex geometry of the dendrites along with their cable properties.

In this study we examine the importance of AC signaling, as an indirect estimate of the influence of back-propagating action potentials, on nonlinear firing patterns. The spatial frequency-response of the passive dendrites in anatomically reconstructed motor neurons was first characterized for an AC signal with a fixed frequency. We expanded our previous reduced modeling framework for DC-signaling (hereafter referred to as a DC-RM) to incorporate the characterized AC signaling property of the dendrites (the expanded reduced model is referred to as a DC/AC-RM). Active membrane mechanisms were added to the DC/AC-RM using a modified Morris-Lecar formulation. We demonstrate how the AC signal attenuation influenced nonlinear firing behaviour in the model motor neuron. Finally we assess how bistable behaviour of the DC/AC-RM compared with the dendritic signaling properties measured directly from anatomically-reconstructed motor neuron models.

## Methods

### Anatomical Neuron Models

The anatomical data of five type-identified cat α–motor neurons were downloaded from http://NeuroMorpho.Org (Archive name: Burke) [22]. The individual anatomical data were translated into the NEURON simulation environment [23] using the Import3D tool and soma geometry was corrected to match the dimensions previously reported [24]. We used the non-uniform specific membrane resistivity (R_m_ Ω⋅cm^2^), cytoplasmic resistivity (R_a_) of 70 Ω⋅cm and a specific membrane capacitance (C_m_) of 1 µF/cm^2^. As previously reported (Table 1 in [21]), the electrotonics (i.e. input resistance, system time constant) of all passive anatomical models were well matched to those experimentally estimated from the linear portion of the steady current-voltage curve and current impulse response near the resting membrane potential before the nonlinearities (presumably contributed by active conductances) occurred.

**Table 1:** Abbreviations.

VGIC	Voltage Gated Ion Channel
PIC	Persistent Inward Current
DDVA	Direction Dependent Voltage Attenuation
AC	Alternating Current
DC	Direct Current
DC-RM	Reduced Model with DC attenuation
DC/AC-RM	Reduced Model with both DC and AC attenuation
VA_SD_^DC^	Voltage Attenuation factor from Soma to Dendrites with DC input
VA_DS_^DC^	Voltage Attenuation factor from Dendrites to Soma with DC input
VA_SD_^AC^	Voltage Attenuation factor from Soma to Dendrites with AC input
CI	Characteristic Index
TTP	Time To onset of Plateau potential
TES	Time to End of Somatic spiking
DSF	Difference in Spiking Frequency

### Frequency Response Analysis

The frequency response (i.e. amplitude ratio and phase shift) [25] of the passive dendritic system was determined as a function of both signal frequency (ω_f_) and path length (D_path_) from the soma. The spatial variations of frequency response over the dendrites were first characterized with the constant ω_f_, then the response to AC signals with various ω_f_ (i.e. frequency vector) was evaluated at a fixed D_path_ of 300 µm, where the calcium PIC channels are believed to be concentrated on the dendrites [9], [15], [16], [17], [18]. For the spatial frequency response analysis, action potentials propagating into the dendrites were represented with a sinusoidal wave (i.e. AC) with the characteristic frequency (ω_f,C_) of 250 Hz. The frequency was selected because the average spike width for motor neurons is about 2 ms [26] and assuming the spike represents half a period of a sinusoidal input, i.e. 4 ms period is equivalent to 250 Hz. Applying this AC signal to the soma of the anatomical models, the characteristic frequency response was calculated from the soma to all individual points over the dendritic trees as a function of D_path_ using the Impedance class in NEURON software [23]. The amplitude ratio and phase shift data were plotted as a function of D_path_ and curve fit to represent the overall response. To compare frequency response in different types of anatomical motor neuron models, the amplitude (VA) and phase (Φ) response at a particular distance from the soma were plotted as a vector (i.e. Nyquist plot) with respect to D_path_ in the complex domain. The real (x) and imaginary (y) component of the frequency-response vector was calculated as,
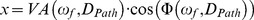
(1)

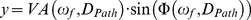
(2)The distance and angle of the individual points from the origin in the complex plane correspond to the amplitude and phase response of the dendrites measured at the specific D_path_.

The new DC/AC-RM approach was validated by comparing the frequency response between the reduced and anatomically reconstructed models at the fixed D_path_ (i.e. 300 µm) over a range of ω_f_ (0–5000 Hz).

### Reduced Neuronal Modeling

**Figure 1:**
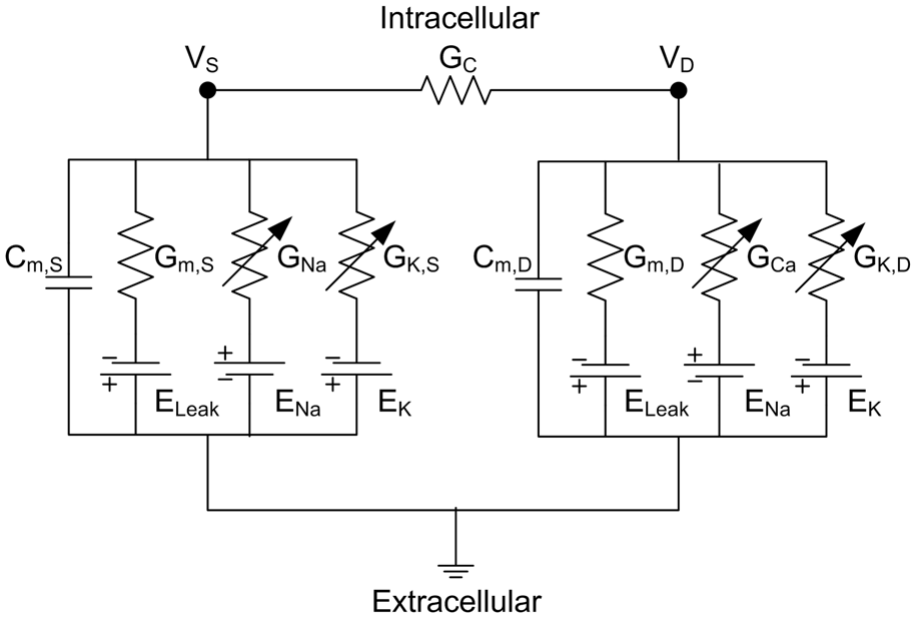
Equivalent circuit representation of a DC/AC-RM. The somatic (left) and dendritic (right) compartments are connected by a coupling conductance, *G_C_*. A key difference between DC/AC-RM and DC-RM is that the membrane capacitance is not uniform. Maximum conductances for the active membrane properties based on the dimensionless Morris-Lecar equations are *G_Na_* for sodium current, *G_K,S_* for somatic potassium current, *G_Ca_* for calcium current and *G_K,D_* for dendritic potassium current. Reversal potentials are *E_Na_*, *E_Ca_*, *E_K_*, and *E_Leak_*. Passive membrane properties are *C_m,S_* and *C_m,D_* for somatic and dendritic membrane capacitances, *G_m,S_* and *G_m,D_* for somatic and dendritic membrane conductances, and *G_C_* for coupling conductance. *V_S_* and *V_D_* are membrane potentials in the somatic and dendritic compartments.

The original DC-RM framework was expanded to retain AC properties by allowing membrane capacitance in the two compartments to be independent. The expanded modeling framework (i.e. DC/AC-RM) is a conductance based model consisting of two compartments coupled by a single conductance. The equivalent circuit of a DC/AC-RM is presented in Figure 1.

#### Passive electrical properties

Applying Kirchhoff’s current law to Figure 1, the equations governing the passive dynamics of the DC/AC-RM are,
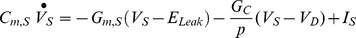
(3)

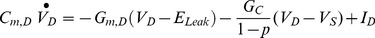
(4)where *V_S_* and *V_D_* are membrane potentials in the soma and dendrite. *p* is the morphological factor defined by the ratio of somatic to total surface area. *I_S_* and *I_D_* are injected currents normalized by somatic and dendritic surface areas. *E_Leak_* is the equilibrium potential for a leak current. *G_m,X_* and *C_m,X_* are specific membrane conductance and capacitance where *X* is *S* or *D* depending on the soma or the dendrites compartment. *G_C_* is coupling conductance between the soma and the dendrite.

The equations for the cable parameters (i.e. *G_m,S_*, *G_m,D_*, *G_C_*, *C_m,S_* and *C_m,D_*) in Eq. (3) & (4) were analytically determined by solving the inverse problem using only the essential biophysical properties measured from one of the anatomical models: input resistance (R_N_), system time constant (τ_m_), and three voltage attenuation factors depending on signal direction and type (soma-to-dendrites with DC (VA_SD_^DC^) and AC (VA_SD_^AC^), and dendrite-to-soma with DC (VA_DS_^DC^)). The values of the three soma-dendrite signaling properties and *p* were determined at a particular distance (D_path_) from the soma for the separation of two compartments. The full set of equations for model parameters and essential system properties are given in Methods S2.

#### Active electrical properties

Membrane excitability in the soma and dendrites was classified using the nonlinear dynamical systems theory [27]. The spiking at the soma (Eq. (3)) was generated via a Hopf bifurcation mechanism mediated by fast Na^+^ (I_Na_) and delayed-rectified K^+^ currents (I_K,S_). The plateau potentials at the dendrites (Eq. (4)) were evoked via a saddle-node bifurcation mechanism mediated by voltage- and time-dependent L-type Ca^2+^ (I_Ca_) and delayed-rectified K^+^ currents (I_K,D_). The somatic membrane excitability was formulated using the Morris-Lecar model [28]. The Morris-Lecar mechanism was modified for the dendritic membrane by making inward current time dependent instead of instantaneous. After the passive membrane properties were solved, the active currents ∑I_A,S_ (I_Na_+ I_K,S_) and ∑I_A,D_ (I_Ca_+I_K,D_) were added to the somatic and dendritic compartments (Figure 1). The dynamics of individual active currents were governed by following conductance based equations,
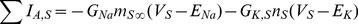
(5)

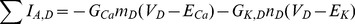
(6)All maximum conductance (i.e. *G_Na_, G_K,S_, G_Ca_* and *G_K,D_* ) and equilibrium potential (i.e. *E_Na_, E_Ca_* and *E_K_*) values in Eq. (5) and (6) were adopted from the bistable DC-RM, so that the bifurcation structure for the nonlinear firing behaviour was conserved in the DC/AC-RM. Bifurcation analysis demonstrating these dynamics is presented in Results. Equations for the gating variables (i.e. *m_S∞_, n_S_, m_D_* and *n_D_*) depending on voltage are given in Methods S1, which includes a full set of system equations and parameter values used in the current study.

### Simulation

We used triangular current stimulation (peak current intensity of I_S_ = 2.5 at time of T = 1350) to the somatic compartment as input to the system. During this triangular current stimulation, firing frequency-current intensity relationship was evaluated to investigate the nonlinear firing behaviour of the new reduced model (i.e. DC/AC-RM) that was induced by the activation of plateau potentials in the dendrite. To facilitate the process of identifying nonlinear firing patterns of the DC/AC-RM during simulations, three characteristic indexes (CIs) were used [20]: Time To onset of Plateau potential (TTP), Time to End of somatic Spiking (TES) and Difference in Spiking Frequency (DSF). “Bistable firing” behaviour, characterized by counter-clockwise frequency hysteresis along with sustained firing resulting from the delayed onset and offset of plateau potentials in the dendrites during the triangular current stimulation [1], [2], was operationally defined by all three indexes having positive values (i.e. TTP>0, TES>0, and DSF>0). Positive values for TTP, TES and DSF represent delayed onset of the plateau potential in the dendrites, sustained firing during the down phase of current stimulation, and higher firing rates during the falling than rising phase of current stimulation at the current threshold for initial spike on the ascending phase respectively. This constraint inference method was validated by directly confirming the bistable firing pattern of the DC/AC-RM that was identified by the positivity of all CIs. Numerical bifurcation analysis of the DC/AC-RM was first conducted for three representative AC signal attenuations. Then the simulations were done with the DC/AC-RM keeping the active properties constant and independently varying the three biophysical signaling parameters (i.e. VA_SD_^DC^, VA_DS_^DC^ and VA_SD_^AC^) from the default value. We evaluated the three characteristic indexes at each location of the three dimensional parameter space, where the location (x, y, z) is defined by the value (VA_SD_^DC^, VA_DS_^DC^, VA_SD_^AC^). The solution space for “bistable firing” was defined as the volume where all three characteristic indexes were positive. We predicted that if the bistable firing was predominantly determined by the DC voltage attenuation parameters, then the volume of solution space would not change as a function of VA_SD_^AC^. If the bistable solution space was completely defined by the DC signaling parameters (VA_SD_^DC^ and VA_DS_^DC^) then the DC/AC-RM would not be needed. All abbreviations used in the present study were presented in Table 1.

## Results

In order to test our hypothesis that the AC signal back-propagation of the passive dendrites plays a role in nonlinear firing behaviour of motor neurons, the present study was done via two steps. We first showed how to derive cable parameters of the reduced model that retained the frequency response of fully reconstructed motor neurons with passive membrane properties (first three sections). Then we demonstrated using the new reduced model with dendritic AC signaling that the AC voltage attenuation indeed affected the nonlinear dynamics of the cell, determining the threshold for activation of the plateau potentials in the dendrites (later three sections).

### Spatial Frequency Response of the Anatomically Reconstructed Models

**Figure 2:**
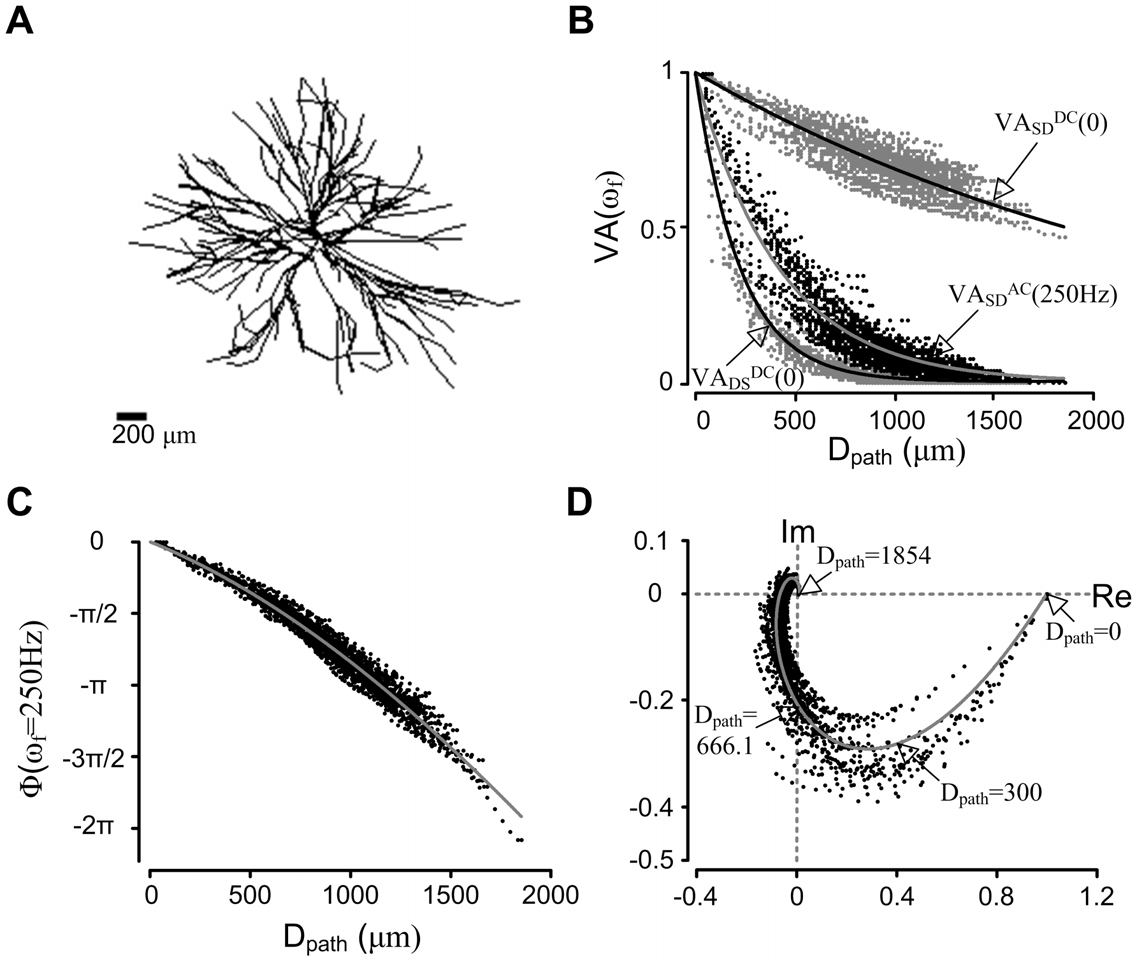
Spatial frequency response of an anatomically reconstructed motor neuron model. (**A**). Morphology of a representative motor neuron. (**B**). Amplitude response (VA): the voltage attenuation data (VA_SD_^AC^(250 Hz) in the middle black dots) for the soma-to-dendrite AC signal with the fixed frequency, ω_f_ = 250 Hz, was superimposed on that for the DC signals in the same direction (VA_SD_^DC^(0) in the top gray dots) and in the opposite direction (VA_DS_^DC^(0) in the bottom gray dots) as a function of the path length (D_path_) from the soma. Each data set was fitted with a single exponential function: e^−Dpath/η^. The η values were 2678.7 for VA_SD_^DC^, 420.1 for VA_SD_^AC^ and 225 for VA_DS_^DC^. (**C**). Phase response (Φ): the phase delay data (black dots) are shown as a function of D_path_, fit with a cubic polynomial function (6.8*10^−11^*D_path_^3^–9.2*10^−7^ *D_path_^2^–0.0018*D_path_, gray line). Negative radian values indicate a phase lag with respect to the input signal. (**D**). The vector representation of distance dependent frequency response in the complex plane consisting of Imaginary (Im in the ordinate) and Real (Re in the abscissa) axes. The imaginary and real component of a vector pointing to the individual data points were calculated using Eq. (1) & (2) (Note: only the end point of the vector is shown; black dots). The distance and angle of the individual vectors from the origin correspond to the amplitude and phase response of the dendrites measured at the specific D_path_ in (**B**) and (**C**). The curve through the data was determined by the lines fit to data in panels (**B**) and (**C**), transformed by Eq. (1) & (2).

For the first step (i.e. fitting the reduced model to AC signal propagation of the dendrites) of this study, the distributed frequency response (amplitude and phase) of the anatomically reconstructed motor neuron models was first characterized along the length of the dendrites applying an AC signal (250 Hz) to the soma. Figure 2 illustrates a representative frequency response of a type-identified anatomical motor neuron model. The four other types of motor neuron models showed qualitatively similar results (Figure S1). Figure 2(A) shows the anatomy of one of the anatomical models and the amplitude (or voltage) attenuation of the input signal that is caused by low pass filtering effects of the passive dendrites is shown in Figure 2(B). The amplitude of the AC signal decayed exponentially with increasing distance from the soma, as predicted from passive cable theory. However, the AC attenuation data was not clustered as in the DC case up to D_path_  = 500 µm, but rather separated into two clusters. This result indicates greater variance in bioelectric impedance than input resistance between individual dendritic trees attached to the soma. The degree of voltage attenuation was more severe with the AC (ω_f_ = 250 Hz) than DC (ω_f_ = 0 Hz) signal for both directions (i.e. soma to dendrites or vice versa). Similar to the DC case, the AC amplitude attenuation from the dendrites to soma (i.e. VA_DS_^AC^, not shown) was greater than that in the opposite direction (i.e. VA_SD_^AC^). The data were fit with a single exponential function (solid black line) to quantify the rate of amplitude attenuation with a decay constant (η). The η was also used in our reduced modeling approach to estimate the degree of signal attenuation at the specific D_path_ from the soma.

Figure 2(C) shows the phase lag of the AC input signal along the path of the dendrites. As D_path_ approached the end of the dendrites, the phase lag increased from 0 to –2π radians. The distance at which the signal became out-of-phase (i.e. –π or 2 ms lag with 250 Hz signal) was relatively far from the soma (i.e. D_path_  = 1200 µm). Even at the most distal branch terminal of the dendritic trees (i.e. D_path_  = 1854 µm), the signal phase was only delayed by approximately –2π radians or 4 ms. The overall phase response data did not decrease linearly unlike linear prediction of phase response with passive cable theory and was better fit to a cubic polynomial function (solid line) as a function of D_path_.

Figure 2(D) demonstrates the overall frequency response including both the amplitude ratio and phase shift in the complex domain as a function of D_path_. It was clear from Figure 2(B) & 2(C) that the amplitude of the AC signal decreased more rapidly than the phase response as D_path_ increased from 0 to 1854 µm. The overall shape of the spatial frequency response (Figure 2(D)) was similar to that of a first-order system response. For D_path_  = 300–500 µm, the current best estimate for the location of calcium dependent PIC channels, the average amplitude of the AC signal attenuated by 71% whereas the average phase lag was only 13% (i.e. 0.26π or 0.52 ms lag). This result indicates that phase lag will have minimal effects on the onset timing of the PIC channels.

### Expansion of Two-compartment Modeling Framework

Having characterized the spatial frequency response of the complex dendrites, the amplitude attenuation of the AC signal turned out to be much more sensitive to the distance from the soma than the phase lag. Based on this finding, we expanded our previous two-compartment model (i.e. DC-RM) with only DC signal propagation to retain the AC amplitude attenuation. For analytical purpose, we released the uniformity of the membrane capacitance assumption in the DC-RM and derived equations for five unknown cable parameters (i.e. *G_m,S_*, *G_m,D_*, *C_m,S_*, *C_m,D_*, *G_C_* in Methods) from equations for five biophysical properties of complex anatomical models (i.e. r_N_, τ_m_, VA_SD_^DC^, VA_DS_^DC^ and VA_SD_^AC^ where r_N_ is the input resistance normalized with somatic surface area).

The equations for *G_m,S_*, *G_m,D_* and *G_C_* were identical to those derived for the DC-RM since *G_m,S_*, *G_m,D_* and *G_C_* were perfectly constrained by r_N_ and DC attenuation properties (i.e. VA_SD_^DC^ and VA_DS_^DC^). Thus we derived here the equations of *C_m,D_* and *C_m,S_*, first for *C_m,D_* from the equation of VA_SD_^AC^ and then for *C_m,s_* from the equation of τ_m_. The equations for DC model parameters (i.e. *G_m,S_*, *G_m,D_* and *G_C_*) are given in Methods S2 with all equations for the DC-RM.

Defining *V_S_* and *V_D_* as the voltage deviations from resting membrane potential in the soma and dendrites, Eq. (4) can be rewritten in the form of,
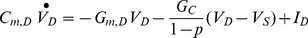
(7)Applying Laplace transformation to the Eq. (7) with *I_D_* = 0, the general equation for the voltage attenuation from the soma to the dendrites is obtained as a function of a complex variable s,
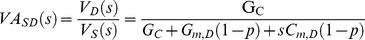
(8)where VASD represents the amplitude attenuation of the AC input signal propagating from the soma to the dendrites.

Substituting jω for s in Eq. (8), the frequency response of the attenuation equation (Eq. (8)) as a function of the frequency (ω) of the AC input signal injected to the soma is,
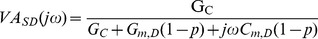
(9)The amplitude response corresponds to the magnitude of the complex number in Eq. (9) given a particular input signal frequency (*ω*),
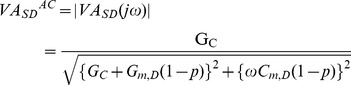
(10)The analytical expressions of the amplitude attenuation for both DC and AC signals can be derived from Eq. (10). When ω is zero Eq. (10) becomes identical to the soma-to-dendritic voltage attenuation (i.e. VASDDC) for DC input (Methods S2). The comparison of DC and AC attenuation equations analytically confirms the result that VASDDC (or VASDAC (ω = 0)) is larger than VASDAC at all distances from the soma (Figure 2(B)).

Rearranging Eq. (10) to get the equation for the dendritic membrane capacitance (*C_m,D_*),
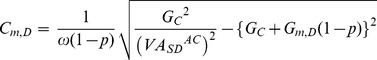
(11)
*C_m,D_* has been analytically determined from the equation of the VA_SD_^AC^ given ω and D_path_. Note that other passive membrane parameters (i.e. *G_m,D_* and *G_C_* in Eq. (11)) are predetermined given R_N_ and dendritic DC signal attenuations (VA_SD_^DC^ and VA_DS_^DC^) measured at a specific D_path_ from the soma.

The equation for the membrane capacitance in the soma (*C_m,S_*) was derived from the equation for the system time constant (τ_m_). Starting with *V_S_* and *V_D_*, the system Eq. (3) & (4) were rearranged into matrix form to get the system matrix,
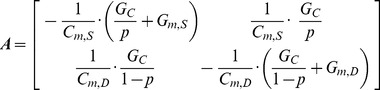
(12)In two-compartment models, there are two membrane time constants that can be calculated analytically by finding the eigenvalues of the system matrix **A**, and confirmed by the peeling technique [29]. The characteristic equation for the system matrix **A** is quadratic in a single scalar variable λ (i.e. the eigenvalue),
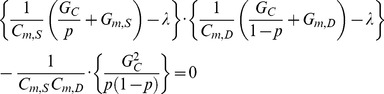
(13)Since Eq. (13) is a second-order polynomial function, it has two solutions or eigenvalues (i.e. λ1< λ2). 1/λ1 represents the membrane time constant (τm) governing the slowest response of the membrane potential to current pulse injected at the soma, whereas 1/λ2 represents an equalizing time constant governing the rapid response of the membrane potential due to the spread of current out to the dendrites [29]. The simplified expression for system time constant (τm) consists of three sub expressions,



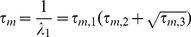
(14)

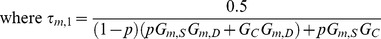


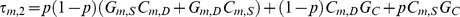





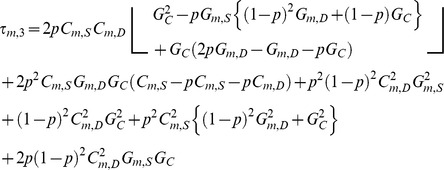



Given that the system time constant is a predetermined passive system property, the equation for the *C_m,S_* was derived from Eq. (13) to give,
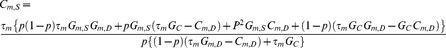
(15)It should be noted that the *C_m,s_* ***must*** be the last calculation in the DC/AC-RM because it includes all other passive membrane parameters (*G_m,S_*, *G_m,D_*, *G_C_* and *C_m,D_*).

### Frequency Response of the DC/AC-RM

Prior to adding active membrane mechanisms for producing nonlinear firing of the DC/AC-RM, we validated the new reduced modeling approach and determined if explicitly adding AC frequency response added necessary features to the original DC-RM. The frequency response of the DC/AC-RM was compared to the corresponding anatomical motor neuron model and DC-RM at the same distance from the soma (D_path_  = 300 µm), for signal frequencies that were not used in developing the new modeling framework because the action potentials propagating to the dendrites have a wide range of frequency components.

Starting with Eq. (8) and adding the passive electrotonic properties we rewrite the equation as a general first-order transfer function,
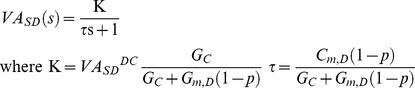
(16)Evaluating Eq. (16) at *s* = *jω*, the frequency response of the first-order system is described by the well-known amplitude and phase equations,
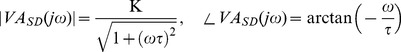
(17)As ω in Eq. (17) increases from zero to infinity, the magnitude of VA_SD_(jω) decreases from a value of K (or VA_SD_^DC^) to zero and the phase lag increases from 0 to – π/2 radians. This analytical estimation of the frequency-response was simulated for both DC-RM (C*_m,S_* = C*_m,D_* = C_*m*_) and DC/AC-RM (C*_m,S_*≠C*_m,D_*), and compared to their corresponding anatomical model at different input signal frequencies (ω).

**Figure 3:**
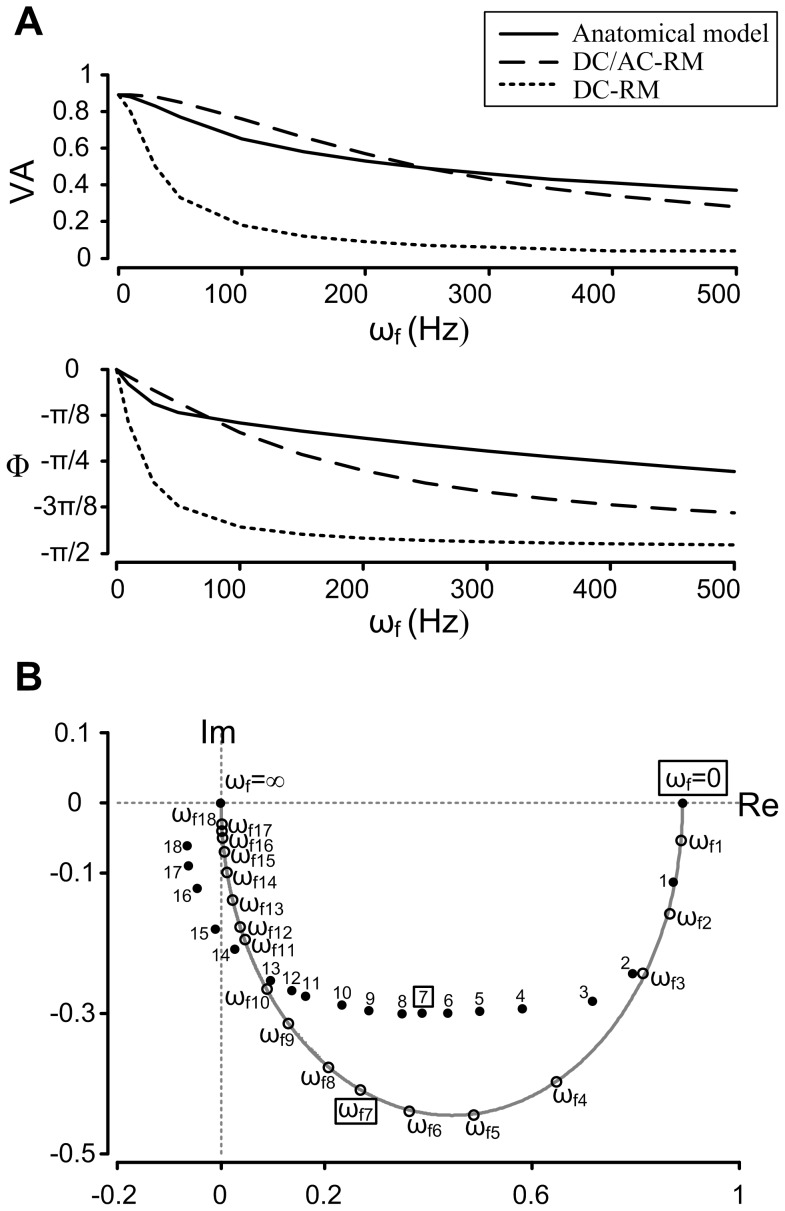
Comparison of the frequency response between the DC/AC-RM, DC-RM and anatomical motor neuron model. (**A**). Bode plots: amplitude (VA) and phase (Φ) response as a function of input signal frequency (*ω*). (**B**). Nyquist plot: integrated representation of frequency response in the complex domain consisting of Imaginary (Im) and Real (Re) axis. The frequency response of the DC/AC-RM is indicated by open circles and the grey line and anatomical model is by the filled circles. The positions of the individual data points were calculated for eighteen frequencies between *ω* = 0 to infinity: *ω_i_* = {10, 30, 50, 100, 150, 200, 250, 300, 400, 500, 700, 800, 1000, 1500, 2000, 3000, 4000, 5000}, where *i* = 1 to 18. The frequencies for the anatomical case were indicated only by *i*. The gray graph represents the frequency response of the DC/AC-RM for the continuous frequency vector. The frequencies for DC (*ω* = 0) and characteristic AC (*ω_7_* = 250 Hz) signals are highlighted with squares.

Figure 3(A) clearly shows that the frequency response of the DC/AC-RM is much closer to the response of the anatomical model, compared to the DC-RM. At the frequencies used to characterize the DC/AC-RM (*ω* = 0 and 250 Hz), the amplitude attenuation was the same in the DC/AC-RM and anatomical model as expected. However the phase lag, which was not included in the development of the DC/AC-RM, was different. The DC/AC-RM had a phase delay that was 0.11π radians greater than in the anatomical case. Amplitude was more attenuated in the anatomical model for frequencies less than 250 Hz but less attenuated above this frequency. The differences in attenuation were greatest at 100 and 700 Hz (not shown). Similarly the phase lag between the DC/AC-RM and anatomical model was same at 75 Hz. Phase was more delayed in the anatomical model for frequencies less than 75 Hz but less delayed above this frequency. The differences in phase were maximized at 30 and 500 Hz by 0.04π and 0.12π radians. The frequencies (i.e. 120 and 500 Hz) maximizing the differences in frequency response were at least two times bigger or less than the characteristic frequency (i.e. 250 Hz), which indicates the capability of the DC/AC-RM to match the frequency response of anatomical models over a broad range of frequencies.

Figure 3(A) suggests that back propagating action potentials would be larger in amplitude in the DC/AC-RM compared to the original DC-RM. At *ω* = 250 Hz, the amplitude attenuation was seven times less in the DC/AC-RM (VA = 0.49) than the DC-RM (VA = 0.07). This result leads to the critical prediction that the dendrite of the DC/AC-RM might be more excitable due to its better transmission of AC signals, leading to the facilitation of voltage gated ion channel activation in the dendrite.

The overall frequency response (i.e. Nyquist plot) of the DC/AC-RM was compared to that of the complex anatomical model at D_path_  = 300 um over the frequency range between 0 to infinity (Figure 3(B)). The reduced model, indicated by open circles and the grey line, has a similar shape to the data from the anatomical model (filled circles). This result illustrates that the DC/AC-RM, which does not solve for phase lag, is a good approximation of the frequency response of the physiological dendrites with passive membrane properties. The mismatch in the data at the frequency of 250 Hz (*ω_7_*) results from the difference in phase response between two models. It should be noted that the conclusions derived from this frequency response analysis were conserved at all distances from the soma (not shown).

### Nonlinear Firing of the Reduced Model with Dendritic AC Signaling

For the second step (i.e. analysis of AC attenuation effects on nonlinear dynamics of motor neurons) of this study, we first evaluated whether the new reduced model (i.e. DC/AC-RM) can produce all features of nonlinear (i.e. bistable) firing behaviour observed in motor neurons [5], [30]. The five passive parameters (*G_m,S_*, *G_m,D_*, *G_C_*, *C_m,D_* and *C_m,S_*) of the DC/AC-RM were determined to retain the five system properties (*r_N_*, *τ_m_*, *VA_SD_^DC^*, *VA_DS_^DC^* and *VA_SD_^AC^*) obtained from the anatomical model as follows: 1) the default values (*VA_SD_^DC^*  = 0.89, *VA_DS_^DC^*  = 0.26, *VA_SD_^AC^*  = 0.49) of the three voltage attenuation factors were specified at D_path_  = 300 µm using fitting equations to individual voltage attenuation data (Figure 2B), 2) *G_m,S_*  = 5.067, *G_m,D_*  = 0.044 and *G_C_*  = 0.299 were uniquely determined from *r_N_*  = 0.19 and two DC voltage attenuation factors (i.e. *VA_SD_^DC^*  = 0.89 and *VA_DS_^DC^*  = 0.26) using the Eq. (2.5)–(2.7) in Methods S2 and 3) the remaining parameters of the model, *C_m,S_*  = 53.103 and *C_m,D_*  = 53.103, were found to reflect the AC voltage attenuation (i.e. *VA_SD_^AC^*  = 0.49) and *τ_m_*  = 10.4 using Eq. (11) & (15) respectively. The same types and parameters of active channels used in the DC-RM were added to the soma and the dendrite to determine if the DC/AC-RM produced firing patterns similar to the DC-RM (refer to Figure 2 in [20]) in response to triangular and current pulse inputs (see Methods S1 for details of active channels). Both current input protocols have been used extensively in experimental and computational studies on motor neuron firing [5], [30], [31]. The triangular current stimulation has been used to demonstrate the counterclockwise frequency-current hysteresis as well as sustained firing behaviour in motor neurons, whereas alternating current pulses have been used to show the bistable state transitions in the steady-state condition.

#### Hyperexcitable dendrite in the DC/AC-RM

The active membrane parameter values that generated robust bistable or Type IV firing, operationally defined in the present report by coincident positive values of the three characteristic indexes, in the DC-RM were applied to the new DC/AC-RM. Using triangular current stimulation the DC/AC-RM produced Type III firing (i.e. no frequency hysteresis with sustained firing behaviour). The result was interpreted as hyperexcitability of the dendrite, likely resulting from the reduced voltage attenuation of the DC/AC-RM (Figure 3(A)). To generate Type IV (or bistable) firing a voltage-dependent activation time constant (i.e. rate constant underlying activation of Ca PIC, τ_mD_ (V_D_) in Eq. (1.2) in Methods S1) was added to the inward current in the dendrite (originally instantaneous). By adding time-dependent activation, the DC/AC-RM recovered the physiological observation of net inward current prior to the bifurcation resulting in the plateau potential in the dendrite [12], [32], [33]. This indicates that the increase in the dendritic excitability arising from the decreased AC signal attenuation, may result in a reduced current threshold for the plateau potential with excitation of the soma.

#### Nonlinear firing patterns

**Figure 4:**
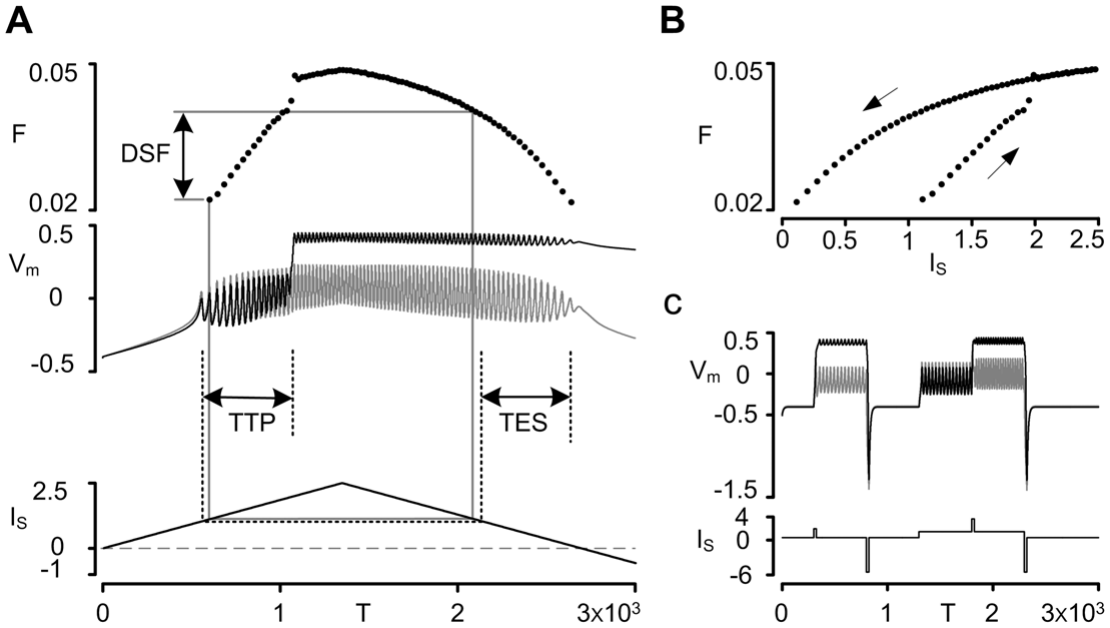
Bistable firing behaviour of the DC/AC-RM. (**A**)**.** Time course of membrane potentials (middle panel: *V_S_* gray, *V_D_* black) during the triangular current stimulation to the soma (bottom) and their instantaneous frequencies (top). Three characteristic indexes for detecting the bistable firing of the model: Time To onset of Plateau potential (TTP), Time to End of somatic Spiking (TES) and Difference in Spiking Frequency (DSF). (**B**). The hysteretic relationship of the frequency responses to current stimuli for the case of (A). (**C**). Switching behaviour of membrane potentials (*V_S_* gray, *V_D_* black) under the briefly depolarizing and hyperpolarizing current stimulation protocol (bottom). V, I, F and T are dimensionless voltage, current, firing rate and time respectively.

Figure 4(A) illustrates the nonlinear firing response of the DC/AC-RM to triangular current injection. As the current stimulation to the soma increased, the somatic membrane potential (*V_S_*) depolarized then transitioned to repetitive spiking. As current stimulation increased there was a nonlinear “jump” to a higher firing frequency that coincided with activation of the plateau potential. The higher firing frequency associated with the plateau potential was sustained during the descending phase of current stimulation. This nonlinear firing behaviour appeared as a counter-clockwise frequency hysteresis in the frequency-current domain (Figure 4(B)). The three characteristic indexes (CIs) were all positive, which by definition indicates the presence of bistable (i.e. Type IV) firing. The *Time To onset of Plateau potential* (TTP) had a positive value because somatic spiking preceded the dendritic plateau onset. As the stimulation decreased toward the value of current threshold determined on the upward phase, the firing frequency remained elevated resulting in a positive *Difference in Spiking Frequency* (DSF). As the current stimulation continued to decrease, the model continued to fire well past the current threshold resulting in a positive value for *Time to End of somatic Spiking* (TES).

Figure 4(C) demonstrates that the model produces two types of the bistable switching behaviour at the same level of steady current stimulation to the soma. At the default steady current level, the transition between the resting state (or stable equilibrium point) and repetitive firing state (or stable limit cycle) was induced by applying brief depolarizing and hyperpolarizing current pulses, respectively. Likewise, a switch between two repetitive firing states of different frequencies was evoked by short depolarizing and hyperpolarizing current pulses added to the depolarized steady current level.

### Influence of Soma-to-dendritic AC Attenuation on the Dynamics of the Reduced Model

The nonlinear firing behaviour of motor neurons has been characterized both theoretically using bifurcation analysis [31] and experimentally measuring firing rates in response to slowly rising and falling current stimulation [2]. Using the DC/AC-RM that produced the bistable firing pattern (Figure 4), we first conducted bifurcation analysis to see how dynamical mechanisms underlying somatic and dendritic excitability were affected by the AC signal attenuation (i.e. VA_SD_^AC^) independently of DC signaling (i.e. VA_SD_^DC^, VA_DS_^DC^). Then, the voltage attenuation effects on frequency-current relationship of the DC/AC-RM was globally investigated with triangular current stimulation to the soma while systematically varying all three voltage attenuation factors over their whole range of values (i.e. 0∼1).

#### Insights from bifurcation analysis

**Figure 5:**
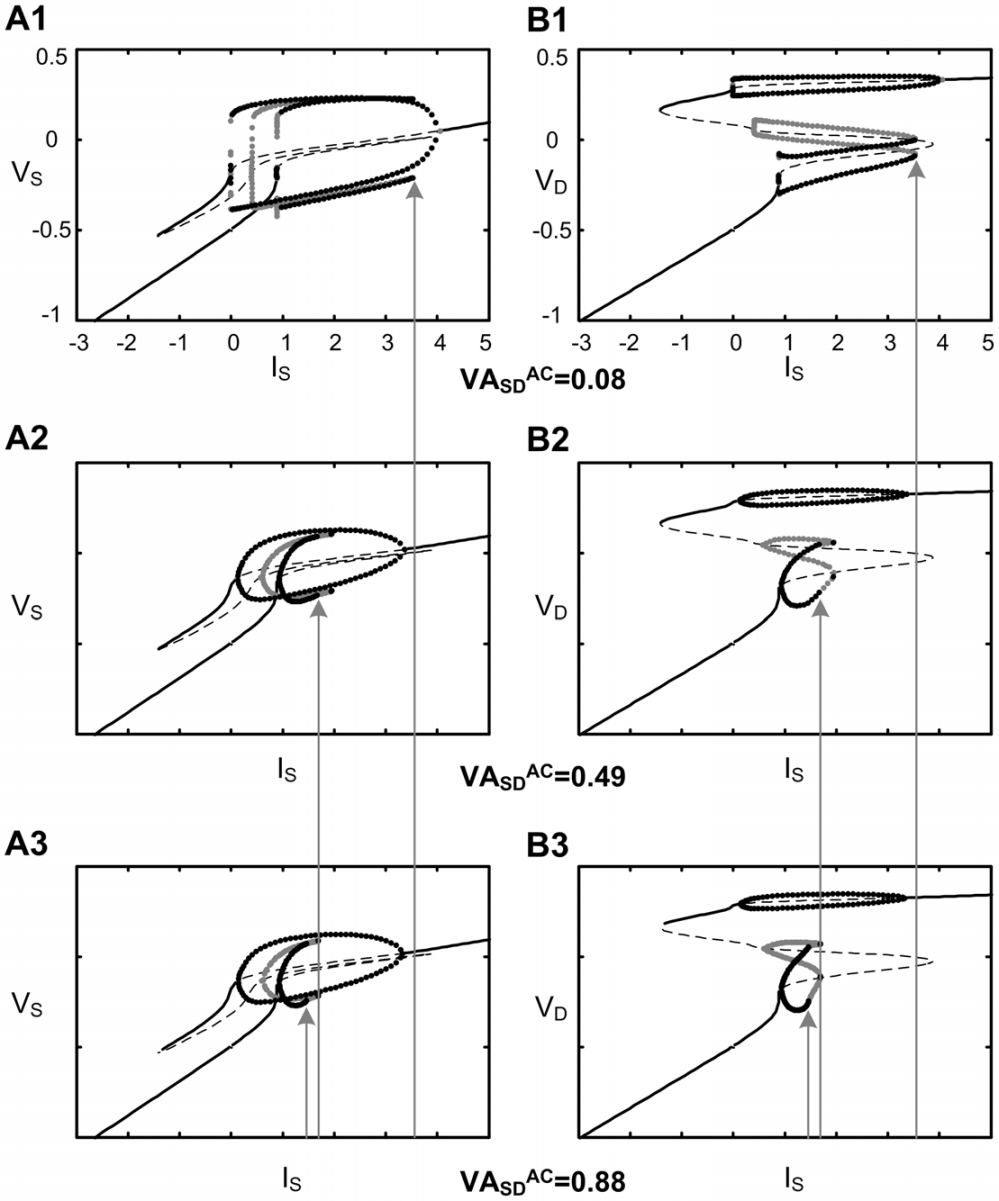
Bifurcation structures at the soma and dendrite of the DC/AC-RM. With increasing AC signal attenuations (VA_SD_^AC^  = 0.08, 0.49 and 0.88) and constant DC signal attenuations (VA_SD_^DC^  = 0.89 and VA_DS_^DC^  = 0.26), the left (soma) and right (dendrite) columns show the solution of the membrane potential as a function of the bifurcation parameter I_S_, i.e. current applied to the soma. For all figures, the stability of fixed points was indicated by black solid line for the stable state and black dashed line for the unstable state. Both stable (black filled circles) and unstable (gray filled circles) limit cycles were outlined with maximum and minimum amplitudes of membrane potential oscillation. The gray solid lines with arrows indicate the current intensity at which the plateau potential is evoked, i.e. saddle-node bifurcation in the dendrite. Note that somatic spiking is initiated at the same bifurcation point regardless of changes in VA_SD_^AC^, and the slope of the V_S_-I_S_ and V_D_-I_S_ curves in the subthreshold region are constant. All model parameters (refer to Method) were held constant, but membrane capacitances were systematically changed according to VA_SD_^AC^ values: C_m,S_  = {19.944, 53.103, 54.583} and C_m,D_  = {2.851, 0.39, 0.039}, where values in curly braces are in the increasing order of VA_SD_^AC^. V and I are dimensionless voltage and current.

Figure 5 shows the correlation between the AC signal attenuation (i.e. VA_SD_^AC^) and dendritic excitability, which was indirectly evaluated by the current threshold for the plateau potential in the dendrite (gray arrows). When VA_SD_^AC^ decreased from the default value of VA_SD_^AC^  = 0.49 to 0.08, the plateau threshold increased about 1.8 times. In contrast, when VA_SD_^AC^ increased to 0.88, the plateau threshold decreased about 0.8 times. This result could be explained by the positive relation between the amplitude of action potentials passively propagated at the dendrite and the value of VA_SD_^AC^ in Figure 5(B1)–(B3). Since the input resistance of the soma (R_N,S_) was a constraint in the DC/AC-RM, the slope of V_S_-I_S_ in the subthreshold region was constant in Figure 5(A1)–(A3). Similarly the input resistance in the dendrite (R_N,D_, indicated by the slope of V_D_-I_S_) could be expected to be constant in the subthreshold region by the relationship derived by Kim and Jones (2011) (i.e. R_N,D_  =  R_N,S_*VA_SD_^DC^/VA_DS_^DC^). The slope of V_S_-I_S_ near the resting membrane potential (i.e. V_S_ = −0.5) was almost identical to the input resistance (*r_N_*  = 0.19) determined by only passive membrane mechanisms of the DC/AC-RM, indicating relatively small contribution of active channels to the resting conductance of the model. The current threshold for spiking at the soma (aka. rheobase), i.e. the Hopf bifurcation, was insensitive to changes in the AC signaling properties. Furthermore, the current threshold for the plateau potential (i.e. the saddle-node bifurcation) at the dendrite was not influenced by the variation of the AC attenuation when the action potentials generated at the soma were blocked (simulated by G_Na_  =  G_K,S_  = 0) (not shown). This result indicates that the change in the current threshold is due to the effects of the AC attenuation, not the local membrane dynamics resulted from change in the membrane conductances (i.e. C_m,S_ and C_m,D_) for setting up the AC attenuation in the new reduced model. A prediction arising from the bifurcation analysis was that solution space for bistable firing in the DC/AC-RM will depend on all three voltage attenuation properties.

#### Solution space for bistable firing

Figure 4 showed that the three characteristic indexes (CIs) were positive in the DC/AC-RM at the default values of voltage attenuation (VA_SD_^DC^  = 0.89, VA_DS_^DC^  = 0.26, VA_SD_^AC^  = 0.49). For the purposes of the present report we have defined bistable firing to be present when the three CIs are positive. To investigate the influence of voltage attenuation on bistable firing of the model, we systematically varied the three voltage attenuation values of the DC/AC-RM. In each parameter set (VA_SD_^DC^, VA_DS_^DC^, VA_SD_^AC^), the positivity of the three CIs was evaluated in response to the triangular current stimulation. Therefore the voltage attenuation values that generate three positive CIs, define a point in the multiparameter space map where the model produced bistable firing behaviour.

**Figure 6:**
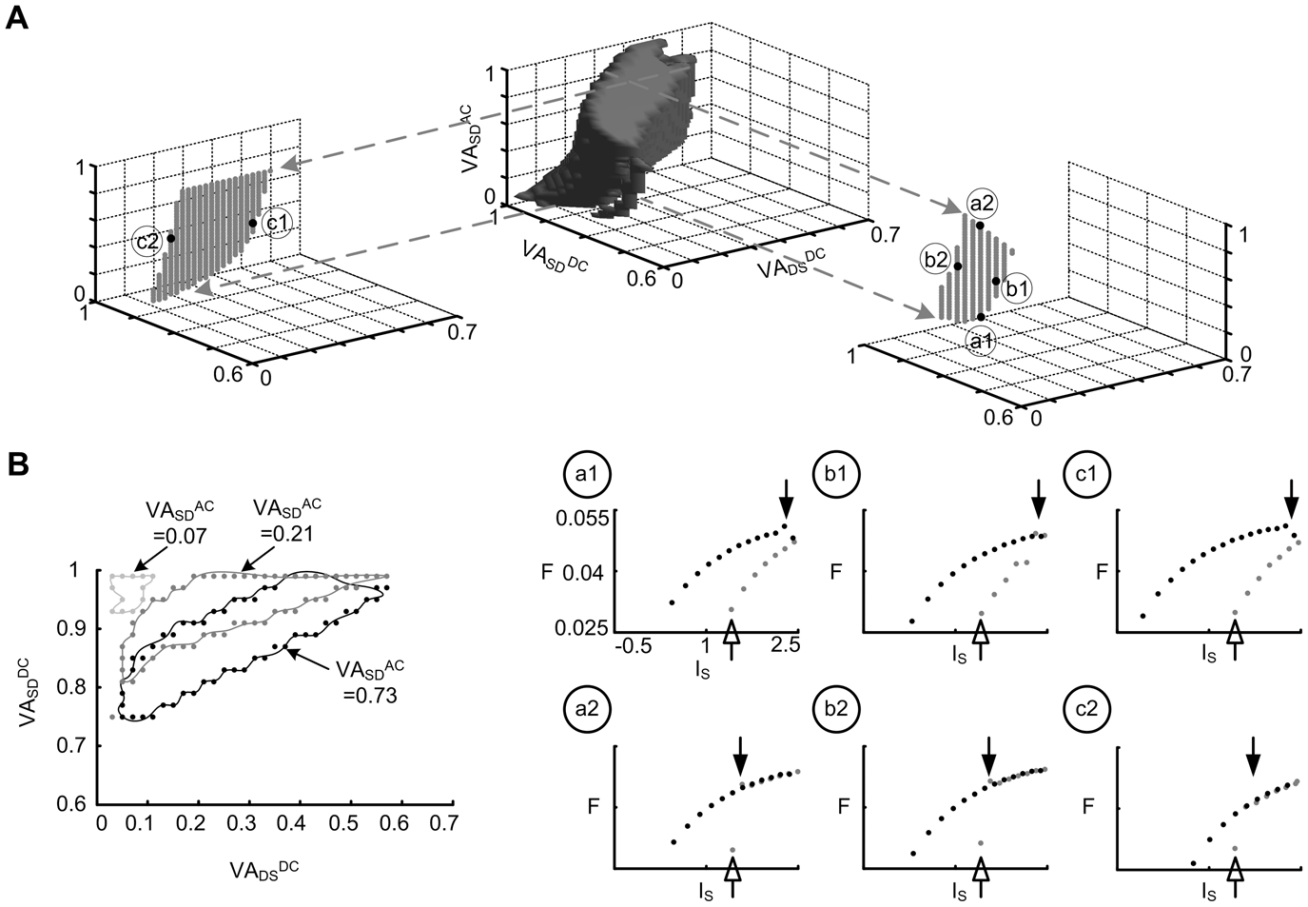
Bistable solution space of the DC/AC-RM. (**A**). The solution parameter sets for the fully bistable firing behaviour of the DC/AC-RM was visualized in the three–dimensional parameter space (VA_DS_^DC^, VA_SD_^DC^, VA_SD_^AC^). Two representative cross section areas of the solution volume at VA_SD_^DC^  = 0.89 (for left gray dots) and VA_DS_^DC^  = 0.26 (for right gray dots) were plotted in separate insets indicated by gray dashed arrows. Six representative solution points were selected along the boundary of two cross section areas to show the firing patterns: a1 (VA_SD_^AC^  = 0.21) – a2 (VA_SD_^AC^  = 0.87), b1 (VA_SD_^DC^  = 0.85) – b2 (VA_SD_^DC^  = 0.95) and c1 (VA_DS_^DC^  = 0.39) – c2 (VA_DS_^DC^  = 0.11). Circled numbers in six frequency (F)–somatic current (I_S_) relationships correspond to the boundary points at each cross section of the solution volume. Open and filled arrows indicate the current threshold (or rheobase) for initiating the spike and the onset of plateau potential in the dendrite respectively. (**B**). Cross sectional areas at different levels of amplitude attenuation of the AC signal (VA_SD_^AC^) on the VA_DS_^DC^–VA_SD_^DC^ plane.

Figure 6(A) shows the distribution of bistable solution points in the three-dimensional voltage attenuation parameter space. Solutions were located in the upper left corner of the VA_DS_^DC^– VA_SD_^DC^ plane, where VA_SD_^DC^ > VA_DS_^DC^. The horizontal cross-section areas of the solution changed as a function of VA_SD_^AC^. Figure 6(B) illustrates the variations of location and size of the horizontal solution space at three representative values of VA_SD_^AC^ (i.e. 0.07, 0.21 and 0.73). The area was largest near 0.73 and smaller at the extremes of VA_SD_^AC^ (i.e. 0.01 and 0.99). The shift in the (VA_DS_^DC^, VA_SD_^DC^) location and size of solution space indicates that the AC voltage attenuation modulates the effects of dendritic input resistance on bistable firing behaviour.

Six representative firing patterns were sampled along the boundary of the bistable solution volume (indicated by circled labels a1–c2, Figure 6(A)). As VA_SD_^AC^ increased from point a1 to a2, the frequency hysteresis significantly decreased due to the earlier onset of the plateau potential (filled arrow in the inset of a2). Similarly, the increase of VA_SD_^DC^ from b1 to b2 and the decrease of VA_DS_^DC^ from c1 to c2 resulted in near simultaneous activation of somatic spiking and the dendritic plateau potential. These results illustrate not only the systematic variation of firing behaviour in solutions defined as fully-bistable, but also the validation of a CIs based constraints inference approach for detecting the bistable solution space of the DC/AC-RMs.

Similar to the DC-RM (see Figure 5 in [20]), three types of firing behaviours were identified outside the solution space based on the F-I curve generated during triangular current stimulation [6]. Type I (linearly overlapping F-I relationship without sustained firing) or II (clockwise F-I relationship with firing rate adaptation) firing behaviour was present in the space below a1 and b1 where the plateau potential was not activated. Type III firing (linearly overlapping F-I relationship with sustained firing) occurred in the space left of c2 and above b2 where the plateau potential was activated simultaneous with somatic spiking. So called “partial bistable firing” [1], [2] and synchronized spiking between the soma and dendrite occurred in the parameter space to the right of c1. Partial bistable firing occurred when the plateau potential was deactivated during the descending phase of current stimulation at values of I_S_ greater than rheobase defined on the ascending phase. Figure S2 contains additional details of firing patterns and the partition of the voltage attenuation parameter space.

### Spatial Relationship of the Bistable Solution Space

**Figure 7:**
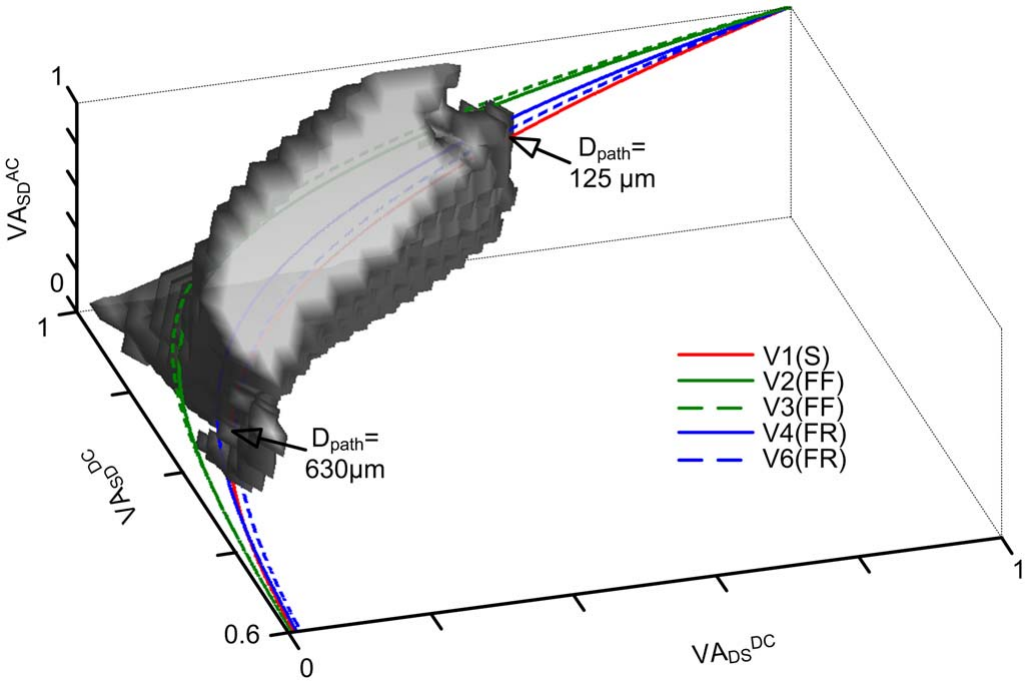
Coincidence of bistable solution space with physiological voltage attenuation properties. The bistable solution space of the DC/AC-RM is outlined by gray patches in the three dimensional parameter space of VA_DS_^DC^, VA_SD_^DC^ and VA_DS_^AC^. To determine the interception area, physiological voltage attenuation values were calculated from anatomically reconstructed motor neurons and superimposed. The lines of physiological voltage attenuation values started from the point of (VA_SD_^DC^  = 1, VA_DS_^DC^  = 1, VA_DS_^AC^  = 1) and ended at the point of (VA_SD_^DC^  = 0.6, VA_DS_^DC^  = 0, VA_DS_^AC^  = 0.04) as a function of the distance from the soma to the tips of the dendrites. The arrows indicate the boundary points of intersection between the solution space and physiological voltage attenuations of the anatomical model V1.

The results from Figure 6 showed that the DC/AC-RM had a large bistable solution space that expanded well beyond the default values for voltage attenuation (VA_SD_^DC^  = 0.89, VA_DS_^DC^  = 0.26 and VA_SD_^DC^  = 0.49). The numerical simulations treated the three voltage attenuation properties as independent, however in physiologically based models the voltage attenuation properties are a function of distance from the soma, D_path_ (Figure 2). Is the theoretical solution space for bistable firing physiologically plausible? To answer this question, we calculated the voltage attenuation properties of five reconstructed motor neurons (Figure 2, Figure S1). Figure 7 shows that the voltage attenuation properties of three motor neurons intersected a large portion of the solution space while the remaining two intersected over a smaller range. The anatomical model V1 that was used in developing the DC/AC-RM intersected the solution space for voltage attenuation values calculated at D_path_  = 125–630 µm. This range of distances matches the hypothesized location of calcium PIC channels (at least 300–500 µm away from the soma) estimated in experimental [15], [16] and theoretical studies [9], [17], [18]. The two motor neurons that intersected the solution space over a limited range are of a different type: Fast-Fatiguable (FF). Experimental data suggested that the probability of bistable firing in these motor neuron types is low [2]. The theoretical solution space of a two-compartment model that is a systematic reduction of complex multi-compartment models includes voltage attenuation values that are physiologically plausible. All these voltage attenuation properties are important contributors to defining what parameter values generate bistable firing behaviour.

## Discussion

We have demonstrated the importance of the retrograde dendritic frequency response to nonlinear firing behaviour using a new reduced modeling framework (i.e. DC/AC-RM). The threshold for activation of plateau potentials and the size of the multiparameter space map generating “fully-bistable” firing, were correlated to the degree of the AC signal attenuation in the direction from the soma to the dendrites, as well as DC attenuation in both directions. Therefore, reduced models examining the nonlinear firing properties should include direction-dependent voltage attenuation (DDVA) parameters for DC and AC signaling. Furthermore we have unequivocally shown that the geometric and passive parameters of our reduced two compartment modeling framework can be directly derived, eliminating the recourse to a phenomenological approach for reduced models of motor neurons.

### Contribution of the Dendritic Signaling to the Nonlinear Firing Behaviour of Motor Neurons

The identification of dendritic parameters governing the activation of PIC channels in the dendrites is crucial to better understand the underlying mechanism of the nonlinear firing in motor neurons. Previous studies using a single parameter such as electrotonic distance in ideal cable models [34] or coupling conductance in two-compartment models [31], have shown that the nonlinear firing behaviour may be produced by dendritic PIC channels that are separated from the soma by a critical electrical distance. However other studies using anatomically reconstructed dendrites have emphasized that PIC channels should be clustered within a limited range of the soma (i.e. 0.62±0.21λ) to generate “fully-bistable” firing behaviour [17]. The discrepancy between the hypothesized localization of dendritic PIC channels from reduced and multicompartment models was shown to be caused by the lack of directional DC voltage attenuation property (i.e. VA_SD_^DC^ and VA_DS_^DC^) in the reduced models [20], [35]. The analysis of the DC/AC-RM in the present study revealed that the AC propagation (VA_SD_^AC^) also modulates the dendritic excitability, implying a critical role for back-propagating action potentials in shaping the nonlinear firing of motor neurons. These findings support the conclusion that at least three direction dependent voltage attenuation (DDVA) parameters (i.e. VA_SD_^DC^, VA_DS_^DC^ and VA_SD_^AC^) along with whole cell properties (i.e. input resistance and membrane time constant) should be considered in reduced neuron models of nonlinear firing behaviour.

### Limitations in Current Modeling Approach

The inferences made about the importance of DDVA for the *in vivo* firing behaviour of mammalian motor neurons, depend on a number of assumptions. The first assumption is that our analysis of six type-identified and reconstructed multicompartment motor neuron models can be generalized to a population of motor neurons with the full range of biophysical properties expected for a motor neuron pool (e.g. R_N_, τ_m_). A second assumption of the proposed modeling framework is that AC voltage attenuation at one frequency, 250 Hz, is sufficient to infer the influence of retrograde propagation of action potentials on plateau potentials, without considering active amplification. Thirdly, we have calculated voltage attenuation, as a function of distance from the soma in the multicompartment models, between two point locations rather than multiple point sources in the dendrites connected to the soma. The fourth assumption we have made is that the Morris-Lecar conductance based models are an adequate approximation of *in vivo* conductances for evaluating the influence of DDVA on nonlinear dynamics of spiking neurons. Each of these assumptions must be considered to assess the limitations of the DC/AC-RM approach.

Estimates of the number of motor neurons connected to mammalian skeletal muscle range from hundreds to thousands [36]. The biophysical properties of the population of motor neurons connected to a muscle are not uniform but vary widely [37]. This heterogeneity of biophysical properties is typically categorized using the notion of motor unit types: Slow-twitch (S)-, Fast-twitch fatigue-resistant (FR)- and Fast-twitch fatigable (FF)-type [38]. Across the range of motor unit types there are differences in dendritic morphology [24], [39] that might give rise to type-specific DDVA. We did not find any overt differences in DDVA in our sample of six type-specific multicompartment models. However this is a small sample size and generalization to a population of hundreds or thousands of motor neurons may be imperfect. We did additional tests with the multicompartment models by varying the somatic input resistance (R_N,S_ from 0.4 to 4.0 MΩ) and measuring the voltage decay constants (η, Figure 2(B)). These tests did not show any evidence for a type-specific dendritic morphology effect on voltage decay constants over and above the effect of R_N,S_. Therefore it seems reasonable at this time to conclude that the DC/AC-RM reduced modeling approach can be generalized to a population of motor neurons.

A secondary finding of these tests was that an essential condition for the analytical solution of the DC/AC-RM passive parameters was found over the full range of R_N,S_. Real values for *C_m,D_* only exist when the following condition is true,
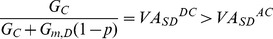
(18)The right hand side of Eq. 18 (derived from Eq. (13)) indicates that a solution for *C_m,D_* only exists when AC signals, at a given distance from the soma, have decayed more than the DC potential (more decay means a smaller value of VA). This is a well-known property resulting from the low-pass filtering characteristics of passive dendritic branches and has been aesthetically presented using the morpho-electrotonic transform [40], [41]. There are conditions in which the right hand side of Eq. (18) may not be true, when voltage-gated ion channels in the dendrites create resonant properties at certain frequencies or mediate active back-propagation of action potentials (for review see Dendrites 2^nd^ edition [42]).

Our second assumption was that characterizing voltage attenuation at 250 Hz in passive dendritic models was sufficient to determine if back-propagating action potentials influence the activation of plateau potentials in the dendrites. While there is some evidence for active back-propagation of action potentials in motor neurons [43], this does not contradict the importance of the underlying passive properties. The active properties are added on top of the foundational passive properties. So solutions for the passive parameters of the DC/AC-RM should still be based on passive dendrite properties. Additional amplifying properties in the dendrite would reinforce our conclusion that retrograde propagation of AC signals is important for explaining the nonlinear dynamics of motor neurons.

The third assumption for the DC/AC-RM was that voltage attenuation was measured between the soma and individual points on the dendrites in the multicompartmental models. This assumption only affects the measurement of VA_DS_^DC^ in the proposed modeling framework. It is reasonable to assume that under certain conditions there may be synchronous, uniformly distributed, current sources on the dendrites at the same distance from the soma (i.e. D_path_). In these conditions it would be more appropriate to measure VA_DS_^DC^ from all points on the dendrites, at the same D_path_, to the soma. The qualitative effect of this change would be to produce an increased sensitivity of somatic voltage to current sources in the dendritic compartment since VA_DS_^DC^ would decrease more slowly with distance from the soma (in contrast to Figure 2(B)). We did not assess this condition in the current study since the purpose of the current study was to determine if VA_SD_^AC^ influenced the activation of plateau potentials. Thus this potential limitation remains to be explored in future research. Our new reduced modeling approach lumping the soma and dendrites into two compartments might not be appropriate in certain cases where the details of individual dendrite properties might be more important, or the voltage attenuation values at the same distance from the soma might not be clustered by the direction and type of input signal so that multiple voltage decay constants (η in Figure 2(B)) might exist for each direction or signal type.

The fourth assumption that may limit the inferences made with the present version of the DC/AC-RM is the use of the Morris-Lecar formulation for ion channel currents. Various types of voltage-gated ion channels have been reported in motor neurons for generating action potentials and plateau potentials [7], [9], [12], [13], [44]. In the current study, all details of active currents were simplified by lumping the many channel types into two: an inward and outward current in each compartment. This choice was made in preference to using Hodgkin-Huxley formalism because it was more appropriate for mathematical analysis. The resulting dynamics of the system retained the emergent properties of threshold and repetitive firing behaviour (i.e. Hopf bifurcation) and plateau phenomenon (i.e. saddle-node bifurcation). Thus our fourth assumption about the lumped representation of active currents seems to be reasonable for the current theoretical study on the influence of the AC voltage attenuation on dynamic behaviour of model neurons. It should be noted that physiological ion channels must be considered in the case where types and kinetics of individual ion channels are important, such as the shape of action potentials including afterhyperpolarization [45] or the modulation of channel activity by neurotransmitters [46]. Further systematic comparison study would be needed to evaluate to what degree the new reduced model (i.e. DC/AC-RM) can match both passive and active (or nonlinear) dynamics of the anatomical model including realistic types, kinetics and distribution of voltage-gated ion channels.

### Comparison with Other Studies

The DC voltage attenuation from the dendrites to the soma has been an important biophysical parameter to explain the contribution of synaptic inputs on the passive dendrites to the subthreshold membrane dynamics at the soma [47], [48], [49]. More recently the AC voltage attenuation from the soma to the dendrites has been experimentally measured to evaluate the facilitation of action potential back-propagation by the voltage-gated ion channels in the dendrites [43], [50], [51]. Since motor neurons have voltage-gated ion channels in the dendrites (e.g. the PIC channels), at least three voltage attenuations should be considered: VA_SD_^DC^ for the current stimulation to the soma, VA_SD_^AC^ for the retrograde action potentials and VA_DS_^DC^ for the plateau potentials in the dendrites.

Anatomical modeling approaches using the morphological details of dendrites implicitly retain all three voltage attenuation properties [9], [17], [18], [52]. However because of the many parameters, these multicompartment models are too complicated for mathematical analysis, as well as being computationally taxing. Reduced modeling approaches, in particular two-compartment models with a lumped soma and dendrite, have been extensively used to theoretically study the influence of the dendrites on nonlinear dynamics in various neurons [11], [31], [53], [54], [55], [56]. In previous two-compartment models, however, passive membrane parameters were phenomenologically determined. Thus the voltage attenuations between the two compartments may not be biophysically plausible and this in turn may lead to misrepresentation of input resistance in the dendrites [20]. This mismatch in the dendritic input resistance may cause significant differences in the output behaviour of reduced models and the original neurons that they were intended to represent [35]. Our new reduced modeling framework bridges the gap between full anatomical and phenomenologically reduced two-compartment modeling studies by analytically deriving the reduced model parameters from multicompartment anatomical models.

The comparison of the DC/AC-RM with both DC and AC attenuations to the DC-RM with only DC attenuations identified collateral roles of AC voltage attenuation in the input-output properties of two-compartment neuron models. The main difference of the two reduced modeling frameworks was the non-uniformity of membrane capacitances (i.e. *C_m,S_* and *C_m,D_* in Eq. (3) & (4)) to retain dendritic AC signaling. The *C_m,D_* in the DC/AC-RM turned out to influence not only the AC signal attenuation (Eq. (10)), but also the *C_m,S_* (Eq. (15)). For the same initial conditions, the *C_m,S_* was ten times larger in the DC/AC-RM than in the DC-RM, resulting in a lower initial firing rate in the DC/AC-RM. Furthermore the linear relationship of firing rate to current intensity prior to the onset of plateau potential and the increase in firing rate upon activation of the plateau potential in the DC/AC-RM qualitatively matched experimental observations [2], [6], [57]. In addition, our simulations of the DC/AC-RM with the instantaneous (i.e. zero activation time constant) activation of plateau potential did not produce physiologically-observed nonlinear firing of motor neurons. The addition of activation time constant (i.e. delayed activation) to the Ca PIC channels in the dendrite was needed for accurate activation profile of plateau potential underlying the nonlinear firing pattern. It was notable that it was very difficult to obtain satisfactory results by varying other activation parameters such as the half-activation voltage or the conductance density of the PIC channels, due to the high sensitivity of the onset of the plateau potential to changes in those PIC activation parameters. This is consistent with the idea of slow kinetics of the PIC channels that has been suggested in previous computational [11], [33] and experimental [12], [32] studies. All these results suggest that the addition of direction dependent AC voltage attenuation is an important parameter for reduced two-compartment modeling frameworks.

### Conclusion

In the important model of Pinsky and Rinzel (1994) it was noted that there was no argument for directly deriving the values of the lumped cable parameters for their two-compartment model from the known passive continuous cable parameters of a more complex model. This study, together with its predecessors, provides the arguments needed for this type of reduction. For the question posed in this study, three direction dependent voltage attenuation properties influenced the emerging dynamics and nonlinear firing behaviour of the motor neuron models. Retrograde transmission of action potentials is a critical factor in forming and stabilizing the bistable firing behaviour of these reduced models. Based on this conclusion, the next step will be to investigate the interaction of the dendritic signal propagation with the active membrane properties on firing dynamics of motor neurons.

## Supporting Information

Figure S1 **Spatial frequency response of type-identified anatomically reconstructed motor neuron models.** Additional four type-identified anatomically reconstructed motor neuron models were adopted from our previous study [21]. All anatomical models with different morphology and whole-cell properties (i.e. R_N_ and *τ_m_*) showed qualitatively similar spatial frequency-response to the AC signal (250 Hz) injected to the soma.
(TIF)

Figure S2 **Partition of the voltage attenuation parameter space.** The different types of non-bistable firing patterns were uncovered in three other subregions outside the bistable space: Type III in the upper-left corner (1), partially bistable & synchronized firing in the upper-middle (2) and the upper-right (3), and Type I & II in the rest (4) space. The lower right corner space represents the region where parameter values for the passive membrane properties are not physiological (i.e. negative cable parameters or non-existence of somatic capacitance to produce system time constant). Representative firing behaviours on each subregion were simulated with triangular current stimulation (I_S_) injected to the soma. 1 to 4 show the non-bistable firing patterns. The somatic and dendritic membrane potentials (V_m_) are indicated by the black and gray colors. Circled letters indicate the corresponding location on the parameter space. T is dimensionless simulation time.
(TIF)

Methods S1 (PDF)

Methods S2 (PDF)
