# Supplementary figures and images for: The Retrograde Frequency Response of Passive Dendritic Trees Constrains the Nonlinear Firing Behaviour of a Reduced Neuron Model
Source: PLoS One. 2012 Aug 20;7(8):e43654. doi: 10.1371/journal.pone.0043654 (PMC3423382; doi:10.1371/journal.pone.0043654)

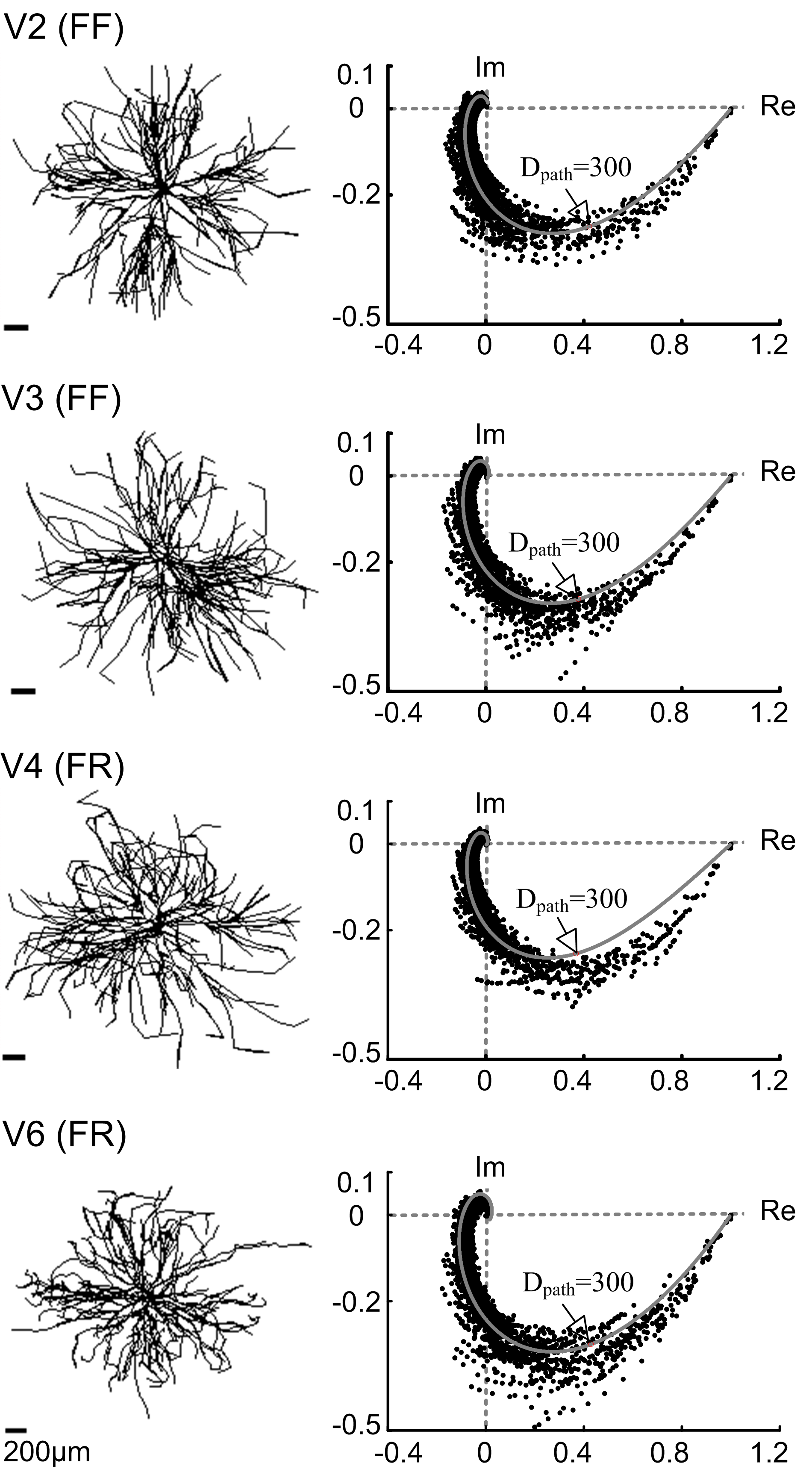

Supplement: Figure S1 — Spatial frequency response of type-identified anatomically reconstructed motor neuron models. Additional four type-identified anatomically reconstructed motor neuron models were adopted from our previous study [21]. All anatomical models with different morphology and whole-cell properties (i.e. RN and τm) showed qualitatively similar spatial frequency-response to the AC signal (250 Hz) injected to the soma. (TIF) [file pone.0043654.s001.tif]

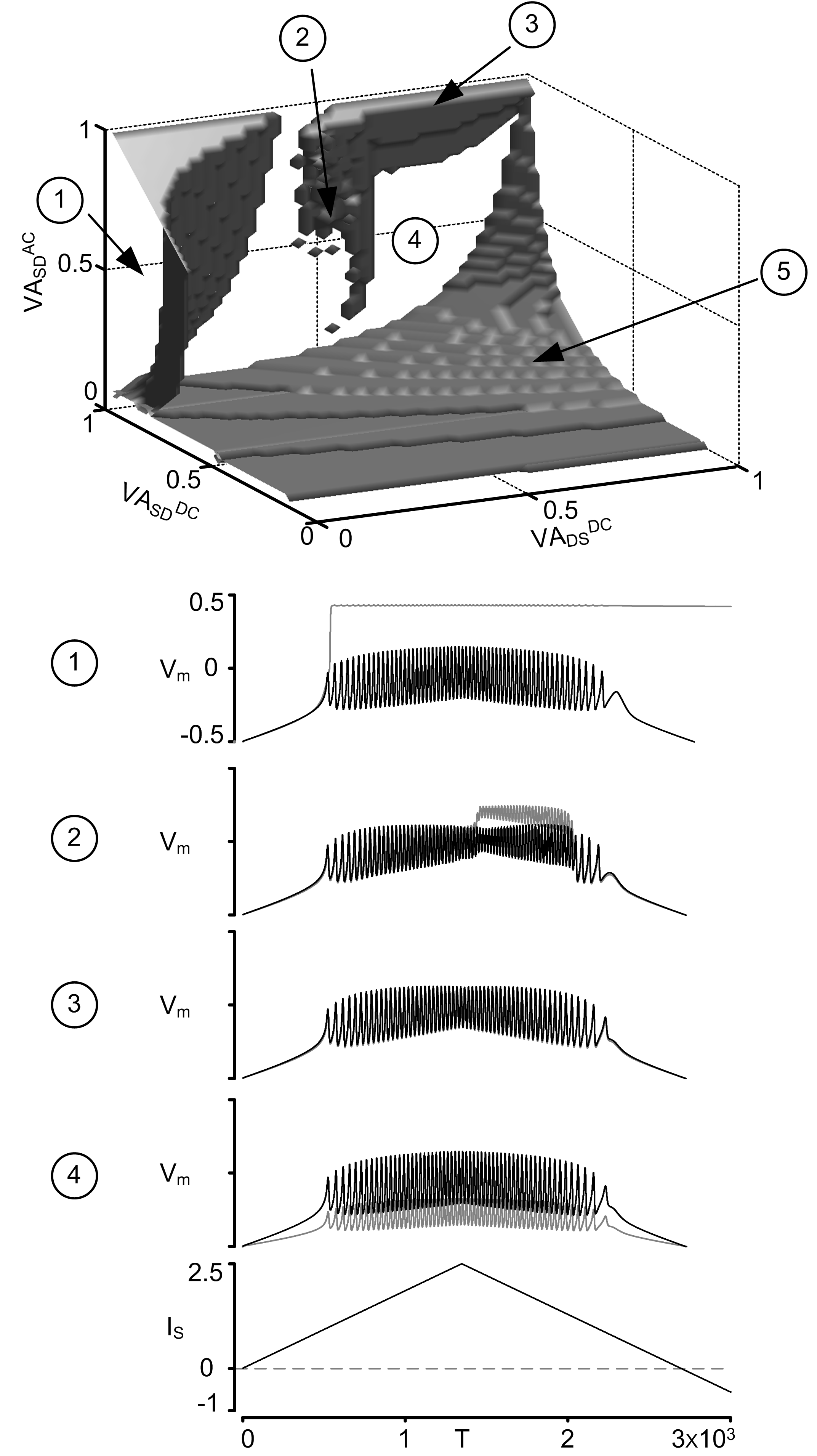

Supplement: Figure S2 — Partition of the voltage attenuation parameter space. The different types of non-bistable firing patterns were uncovered in three other subregions outside the bistable space: Type III in the upper-left corner (1), partially bistable & synchronized firing in the upper-middle (2) and the upper-right (3), and Type I & II in the rest (4) space. The lower right corner space represents the region where parameter values for the passive membrane properties are not physiological (i.e. negative cable parameters or non-existence of somatic capacitance to produce system time constant). Representative firing behaviours on each subregion were simulated with triangular current stimulation (IS) injected to the soma. 1 to 4 show the non-bistable firing patterns. The somatic and dendritic membrane potentials (Vm) are indicated by the black and gray colors. Circled letters indicate the corresponding location on the parameter space. T is dimensionless simulation time. (TIF) [file pone.0043654.s002.tif]
