# Supplementary material for: The Retrograde Frequency Response of Passive Dendritic Trees Constrains the Nonlinear Firing Behaviour of a Reduced Neuron Model
Source: PLoS One. 2012 Aug 20;7(8):e43654. doi: 10.1371/journal.pone.0043654 (PMC3423382; doi:10.1371/journal.pone.0043654)
Supplement: Methods S1 — (PDF) [file pone.0043654.s003.pdf]

Supporting Method 1

The membrane potential dynamics of the DC/AC-RM was governed by the following current-balance equations for somatic and dendritic compartments,

$$C_{m,S}\dot{V}_S = -G_{m,S}(V_S - E_{Leak}) - \frac{G_C}{p}(V_S - V_D) - G_{Na}m_{S\infty}(V_S - E_{Na}) - G_{K,S}n_S(V_S - E_K) + I_S \quad (1.1)$$

$$m_{S\infty}(V_S) = 0.5\left(1 + \tanh\frac{V_S + 0.01}{0.15}\right)$$

$$\dot{n}_S = 0.2\left(\frac{n_{S\infty}(V_S) - n_S}{\tau_S(V_S)}\right) \text{ where } n_{S\infty}(V_S) = 0.5\left(1 + \tanh\frac{V_S + 0.04}{0.1}\right),\ \tau_S(V_S) = \left(\cosh\frac{V_S + 0.04}{0.1}\right)^{-1}$$

$$C_{m,D}\dot{V}_D = -G_{m,D}(V_D - E_{Leak}) - \frac{G_C}{1-p}(V_D - V_S) - G_{Ca}m_D(V_D - E_{Ca}) - G_{K,D}n_D(V_D - E_K) + I_D \quad (1.2)$$

$$\dot{m}_D = 0.2\left(\frac{m_{D\infty}(V_D) - m_D}{\tau_{mD}(V_D)}\right) \text{ where } m_{D\infty}(V_D) = 0.5\left(1 + \tanh\frac{V_D - 0.07}{0.1}\right),\ \tau_{mD}(V_D) = \left(\cosh\frac{V_D - 0.07}{0.1}\right)^{-1}$$

$$\dot{n}_D = 0.2\left(\frac{n_{D\infty}(V_D) - n_D}{\tau_{nD}(V_D)}\right) \text{ where } n_{D\infty}(V_D) = 0.5\left(1 + \tanh\frac{V_D}{0.1}\right),\ \tau_{nS}(V_S) = \left(\cosh\frac{V_D}{0.1}\right)^{-1}$$

where the subscripts of S and D indicate the soma and dendrites. V_S and V_D are membrane potentials in the somatic and dendritic compartment. I_S and I_D are the current density injected at the soma and dendritic compartment. Cable parameter values are $C_{m,S}$=53.103, $C_{m,D}$=0.39, $G_{m,S}$=5.067, $G_{m,D}$=0.044 and G_C=0.299 given the essential biophysical properties measured from the anatomical model (V1 in Figure 2(A)): r_N=0.19, τ_m=10.4, VA_{SD}^{DC}=0.89, VA_{SD}^{AC}=0.49, VA_{DS}^{DC}=0.26, and p=0.168. Maximum conductance values are G_{Na}=11.0, $G_{K,S}$=14.0, G_{Ca}=0.89 and $G_{K,D}$=0.44. Reversal potentials are E_{Na}=1.0, E_{Ca}=1.0, E_K= – 0.7, E_{Leak}=-0.5. All model parameters were non-dimensionalized [1].

Reference

1. Booth V, Rinzel J (1995) A minimal, compartmental model for a dendritic origin of bistability of motoneuron firing patterns. J Comput Neurosci 2: 299-312.
