# Supplementary material for: The Retrograde Frequency Response of Passive Dendritic Trees Constrains the Nonlinear Firing Behaviour of a Reduced Neuron Model
Source: PLoS One. 2012 Aug 20;7(8):e43654. doi: 10.1371/journal.pone.0043654 (PMC3423382; doi:10.1371/journal.pone.0043654)
Supplement: Methods S2 — (PDF) [file pone.0043654.s004.pdf]

Supporting Method 2

Forward and inverse equations for the DC-RM [1]. The system equations are identical to the DC/AC-RM except for the uniform membrane capacitance (i.e. C_m=$C_{m,S}$=$C_{m,D}$). Essential electrotonic properties that constrain the model parameters (i.e. $G_{m,S}$, $G_{m,D}$, C_m, G_C) are input resistance ($r_{N,S}$) normalized by somatic surface area, system time constant (τ_m), and directional voltage attenuations ($VA_{SD}{}^{DC}$ and $VA_{DS}{}^{DC}$) with the DC input. Note that forward equations for $r_{N,S}$, $VA_{SD}{}^{DC}$ and $VA_{DS}{}^{DC}$, and inverse equations for $G_{m,S}$, $G_{m,D}$ and G_C are not influenced in the DC/AC-RM to retain the amplitude attenuation of the AC signals ($VA_{SD}{}^{AC}$). Refer to Eq. (10)-(11) for $VA_{SD}{}^{AC}$ and $C_{m,D}$, and Eq. (14)-(15) for τ_m and $C_{m,S}$ in the DC/AC-RM.

► **Forward equations:**

$$r_{N,S} = \frac{V_S}{I_S} = \frac{p}{G_C}\left(\frac{G_{m,S}}{G_C}p + \frac{G_{m,D}(1-p)}{G_C + G_{m,D}(1-p)}\right)^{-1} \tag{2.1}$$

$$VA_{SD}{}^{DC} = \frac{V_D}{V_S} = \frac{G_C}{G_C + G_{m,D}(1-p)} \tag{2.2}$$

$$VA_{DS}{}^{DC} = \frac{V_S}{V_D} = \frac{G_C}{G_C + G_{m,S}\,p} \tag{2.3}$$

$$\tau_m = R_{\mathrm{eff}} C_m \tag{2.4}$$

$$R_{eff} = \frac{2P(P-1)}{\sqrt{G_C^2 + 2(G_{m,S} - G_{m,D})\cdot(1-2P)\cdot(1-P)PG_C + (G_{m,S}^2 + G_{m,D}^2 - 2G_{m,S}G_{m,D})\cdot(1-P)^2P^2} + P(P-1)(G_{m,S} + G_{m,D}) - G_C}$$

► **Inverse equations:**

$$G_{m,S} = \frac{1 - VA_{DS}{}^{DC}}{r_N(1 - VA_{SD}{}^{DC} VA_{DS}{}^{DC})} \tag{2.5}$$

$$G_{m,D} = \frac{\mathrm{pVA_{DS}}^{\mathrm{DC}}(1 - VA_{SD}{}^{DC})}{(1-p)r_N VA_{SD}{}^{DC}(1 - VA_{SD}{}^{DC} VA_{DS}{}^{DC})} \tag{2.6}$$

$$G_C = \frac{\mathrm{pVA_{DS}}^{DC}}{r_N(1 - VA_{SD}{}^{DC} VA_{DS}{}^{DC})} \tag{2.7}$$

$$C_m = \frac{\tau_m}{R_{\mathrm{eff}}} \tag{2.8}$$

Reference

1. Kim H, Major LA, Jones KE (2009) Derivation of cable parameters for a reduced model that retains asymmetric voltage attenuation of reconstructed spinal motor neuron dendrites. J Comput Neurosci 27: 321-336.
